# Revisiting the Abscopal Effect in the Era of Immuno-Radiotherapy: Mechanisms, Challenges, and Clinical Perspectives

**DOI:** 10.3390/biom16081185

**Published:** 2026-08-14

**Authors:** Enrico Rosa, Maria Vaccaro, Bruno Fionda, Valentina Lancellotta, Gabriele Ciasca, Pierpaolo Dragonetti, Lucia Di Maio, Fabio Marazzi, Francesco Marampon, Monica Mangoni, Maria Antonietta Gambacorta, Marco De Spirito, Luca Tagliaferri

**Affiliations:** 1 Sezione di Fisica, Dipartimento di Neuroscienze, Università Cattolica del Sacro Cuore, 00168 Roma, Italy; 2 UOC Fisica per le Scienze della Vita, Dipartimento di Diagnostica per Immagini e Radioterapia Oncologica, Fondazione Policlinico Universitario A. Gemelli IRCCS, 00168 Roma, Italy; 3 UOC Degenze per la Radioterapia Oncologica, Dipartimento di Diagnostica per Immagini e Radioterapia Oncologica, Fondazione Policlinico Universitario A. Gemelli IRCCS, 00168 Roma, Italy; 4 Department of Radiological Sciences, Oncology, and Anatomical Pathology, Sapienza University of Rome, Policlinico Umberto I, Viale del Policlinico 155, 00161 Rome, Italy; 5 Radiotherapy Unit, Department of Experimental & Clinical BiomedicalSciences, University of Florence, Largo Brambilla 3, 50134 Florence, Italy; 6 UOC Servizio di Radioterapia Oncologica, Dipartimento di Diagnostica per Immagini e Radioterapia Oncologica, Fondazione Policlinico Universitario A. Gemelli IRCCS, 00168 Roma, Italy; 7 Dipartimento di Scienze Radiologiche ed Ematologiche, Università Cattolica del Sacro Cuore, 00168 Roma, Italy

**Keywords:** abscopal effect, immuno-radiotherapy, immunogenic cell death, tumor microenvironment

## Abstract

**Background:** The abscopal effect refers to the clinical response of non-irradiated tumor lesions following localized radiotherapy (RT). Once regarded as a rare phenomenon, it has gained renewed interest in the era of immunotherapy, as RT may promote systemic anti-tumor immune responses. This narrative review summarizes current biological, radiobiological, and clinical evidence on the abscopal effect and highlights translational gaps limiting its reproducibility. **Methods:** Preclinical, translational, and clinical evidence was qualitatively analyzed across seven domains: RT, immunology, and clinical oncology. A structured qualitative gap analysis was used to identify disconnections between biological mechanisms, RT parameters, biomarkers, and clinical outcomes. **Results:** Current evidence supports the biological plausibility of the abscopal effect through immunogenic cell death, antigen and damage-associated molecular patterns (DAMP) release, activation of the cyclic GMP–AMP synthase–stimulator of interferon genes (cGAS-STING) pathway, dendritic-cell priming, and T-cell-mediated responses. However, clinical results remain heterogeneous. Dose, fractionation, irradiated volume, timing, lymphocyte preservation, and interventional RT (modern brachytherapy, IRT) may influence systemic immune activation. Emerging biomarkers, particularly extracellular vesicles (EVs), may help connect radiation-induced biological stress with immune modulation and clinical response. **Conclusions:** The main barrier to clinical translation is the fragmentation of evidence across RT, immunology, and clinical oncology. Integrated translational frameworks combining dosimetry, immune monitoring, EVs-based biomarkers, imaging, and clinical endpoints may improve the interpretation and reproducibility of abscopal responses in immuno-RT.

## 1. Introduction

The abscopal effect is one of the most intriguing and controversial phenomena in modern oncology. Originally described by Mole in 1953 as a biological response occurring outside the irradiated field, the phenomenon was long considered anecdotal and exceptionally rare [1]. Over the past decade, however, the rapid development of immunotherapy and the increasing understanding of radiation-induced immune modulation have renewed scientific interest in the systemic effects of localized radiotherapy (RT).

Traditionally, RT has been regarded as a purely local treatment capable of inducing tumor cell death through direct DNA damage. Growing evidence suggests that ionizing radiation also exerts profound immunological effects. Radiation can induce immunogenic cell death, promote the release of tumor-associated antigens and damage-associated molecular patterns (DAMPs), stimulate dendritic-cell activation, and enhance cytotoxic T-cell priming [2,3]. Through these mechanisms, the irradiated tumor may act as an in situ vaccine, converting local tumor damage into a systemic anti-tumor immune response.

**Figure 1:**
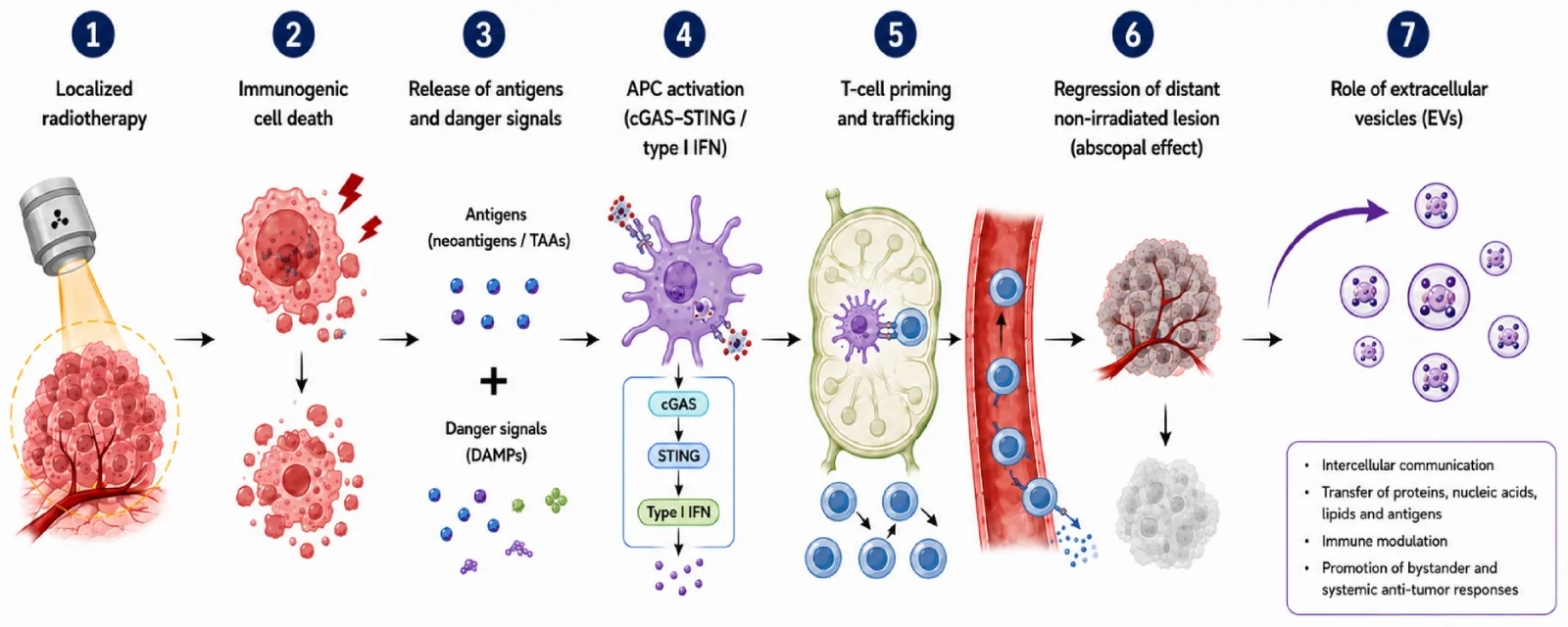
Mechanistic framework of radiation-induced abscopal immune response. See main text for details.

The interaction between RT and the immune system is highly complex and involves several molecular and cellular pathways. Radiation-induced DNA damage may activate the cyclic GMP-AMP synthase–stimulator of interferon genes (cGAS-STING) pathway, contributing to type I interferon production, innate immune activation, and enhanced antigen presentation [4,5,6]. At the same time, radiation can modify the tumor microenvironment by altering cytokine release, promoting immune-cell trafficking, increasing antigen presentation, and influencing immune-checkpoint expression [7]. The biological sequence linking localized irradiation to distant immune-mediated tumor regression is summarized in Figure 1.

These immunological effects have become particularly relevant in the era of immune checkpoint inhibitors. The introduction of anti-PD-1, anti-PD-L1, and anti-CTLA-4 therapies has dramatically changed the therapeutic landscape of several tumors, including melanoma, cutaneous squamous cell carcinoma, and head and neck squamous cell carcinoma [8]. In this context, immune checkpoint blockade may amplify radiation-induced anti-tumor immunity by sustaining T-cell activation, reducing immune escape, and enhancing systemic tumor control.

Despite growing interest, the abscopal effect remains incompletely understood and inconsistently reproducible in clinical practice. One of the major limitations of the field lies in the fragmentation of the evidence. Studies in immunotherapy frequently focus on dosimetric and technical aspects, immunological investigations explore molecular and cellular mechanisms, while clinical oncology studies mainly report treatment outcomes. These domains often evolve independently, with limited methodological integration and minimal cross-disciplinary communication [9].

This disconnect contributes to several unresolved questions. The optimal radiation dose and fractionation capable of maximizing systemic immune activation remain uncertain. The role of irradiated volumes, treatment timing, sequencing with immunotherapy, tissue radiosensitivity, and emerging immune biomarkers is still debated. Furthermore, clinical evidence remains heterogeneous and frequently difficult to compare because of differences in patient populations, treatment strategies, and reporting criteria.

Recent advances in immune-RT (I-RT) have highlighted the importance of adapting RT to immunotherapy rather than simply combining the two treatment modalities, thereby introducing the ABundantia Immunis Localis (SCOPAL) paradigm, in which RT provides a radiation immune boost (RIB) that enhances systemic immune responses.

In head and neck cancer, randomized prospective trials such as JAVELIN Head and Neck 100 and KEYNOTE-412 failed to demonstrate clear survival benefits from adding immune checkpoint inhibitors to standard chemoradiotherapy despite strong radiobiological rationale [10,11]. Conversely, retrospective experiences in skin cancer have reported encouraging outcomes with hypofractionated RT and concurrent cemiplimab, suggesting that smaller irradiated volumes and different fractionation schedules may favor immune activation [12,13].

Collectively, these observations suggest that the discrepancy between preclinical radiobiological premises and clinical outcomes may derive not from the absence of synergy between RT and immunotherapy, but rather from insufficient adaptation of RT parameters to immune-mediated mechanisms [14].

The aim of the present narrative review is therefore to provide an updated synthesis of the biological, radiobiological, and clinical evidence related to the abscopal effect while evaluating the existing gaps between RT, immunology, and clinical oncology. Particular attention is devoted to treatment fractionation, irradiated volumes, timing of combination therapies, tissue radiosensitivity, immune biomarkers, and the potential role of interventional RT (IRT, also referred to as modern brachytherapy) and advanced radiation techniques within future I-RT paradigms.

Therefore, this review proposes an integrated translational framework linking radiation-related parameters, immune activation, tumor microenvironment modulation, and emerging clinical biomarkers to better interpret the current limitations in reproducing the abscopal effect. Rather than considering the abscopal response as an isolated or unpredictable event, this framework aims to contextualize it as the result of a complex interaction between dose, fractionation, irradiated volume, treatment sequencing, lymphocyte preservation, and systemic immune competence. By connecting radiobiological mechanisms with clinically measurable endpoints, including immune monitoring, imaging features, and extracellular vesicle-based biomarkers, this review seeks to clarify why abscopal responses remain clinically heterogeneous and how future immuno-radiotherapy strategies may be more rationally designed to improve their reproducibility.

## 2. Methods

A narrative review methodology was conducted to provide a multidisciplinary overview of the abscopal effect and its relationship with modern immunotherapy strategies. Given the conceptual and methodological heterogeneity of the field, the aim was not to perform an exhaustive systematic synthesis, but rather to integrate preclinical, translational, and clinical evidence relevant to the biological and therapeutic mechanisms underlying systemic radiation-induced immune responses.

The literature considered in this review comprised narrative reviews, translational studies, preclinical studies, retrospective analyses, prospective clinical trials, and selected case reports exploring the interaction between RT and systemic anti-tumor immunity. Particular attention was devoted to studies investigating radiation fractionation, irradiated volumes, immune modulation, sequencing between RT and immunotherapy, and the clinical evidence supporting abscopal responses.

The retrieved literature was qualitatively analyzed according to seven principal domains: RT, immunology, clinical response, tumor microenvironment, extracellular vesicles, in vitro models, and advanced technologies. RT studies primarily focused on treatment delivery, fractionation, stereotactic techniques, IRT, dosimetric considerations, and radiation-induced immune modulation. Immunological studies explored molecular pathways such as immunogenic cell death, dendritic-cell activation, T-cell priming, cytokine release, cGAS-STING signaling, and tumor microenvironment modulation. Clinical investigations evaluated systemic tumor responses, treatment outcomes, and the translational implications of combining RT with immunotherapy.

Subsequently, a structured qualitative gap analysis was performed to evaluate the degree of interaction between these domains and to identify the principal limitations preventing the development of a unified and clinically actionable framework for the abscopal effect. This approach was designed to identify areas of disconnection between biological mechanisms, RT-related parameters, and clinical outcomes, thereby supporting a translational interpretation of the available evidence rather than a quantitative synthesis.

## 3. Results

The literature analyzed in this review confirms that the abscopal effect is a biologically plausible but clinically heterogeneous phenomenon arising from the interplay between RT-induced tumor damage and systemic immune activation. The available evidence can be organized into three major, albeit only partially interconnected, domains. This multidisciplinary structure, together with the principal gaps limiting integration between these fields, is summarized in Table 1.

Preclinical investigations consistently demonstrate that radiation can induce immunogenic cell death through the release of tumor antigens and DAMPs, leading to dendritic-cell activation and CD8+ T-cell priming [15]. Experimental evidence also supports the central role of fractionation in modulating systemic immune responses. Dewan et al. demonstrated that fractionated RT, rather than single high-dose irradiation, was capable of inducing immune-mediated abscopal responses when combined with anti-CTLA-4 therapy [16]. Similarly, recent translational studies suggest that hypofractionation enhances immunogenicity while limiting excessive activation of immunosuppressive pathways [2].

The tumor microenvironment has emerged as a key determinant of treatment response. While radiation can increase antigen presentation and T-cell infiltration, it may also induce immunosuppressive mechanisms such as regulatory T-cell recruitment, myeloid-derived suppressor-cell accumulation, and PD-L1 upregulation [9]. Large irradiated volumes appear particularly detrimental because of radiation-induced lymphopenia and systemic immune suppression [17]. Collectively, these findings support the hypothesis that smaller target volumes and partial irradiation strategies may preserve systemic immune function more effectively than extensive irradiation fields.

Clinical evidence remains heterogeneous and appears to depend largely on tumor type and treatment strategy. Skin cancers and melanoma provide the strongest clinical evidence supporting systemic immune-mediated responses. Recent retrospective studies evaluating concurrent RT and cemiplimab in advanced cutaneous squamous cell carcinoma demonstrated faster and more durable responses without substantial increases in toxicity [12,13,18]. Additional reports involving alpha-emitter modern brachytherapy have also described systemic regression of non-irradiated lesions following localized treatment, supporting the immunogenic potential of high-linear energy transfer (LET) IRT approaches. LET represents the energy deposited by ionizing radiation per unit path length [19].

By contrast, prospective randomized studies in head and neck cancer have produced less encouraging results. The JAVELIN Head and Neck 100 trial failed to demonstrate survival improvement with the addition of avelumab to standard chemoradiotherapy [10]. Similarly, KEYNOTE-412 did not show statistically significant improvement with pembrolizumab combined with concurrent chemoradiotherapy [11]. These disappointing outcomes may be related to large irradiated volumes, conventional fractionation schedules, treatment-related lymphopenia, and inadequate patient selection.

Several studies have emphasized the importance of treatment timing and sequencing. Evidence suggests that neoadjuvant or early integration of immunotherapy may produce stronger immune activation compared with adjuvant administration [20,21]. Radiosensitivity also appears to influence treatment efficacy, as different tissues and tumor histologies demonstrate variable immunogenic responses after irradiation [22].

**Figure 2:**
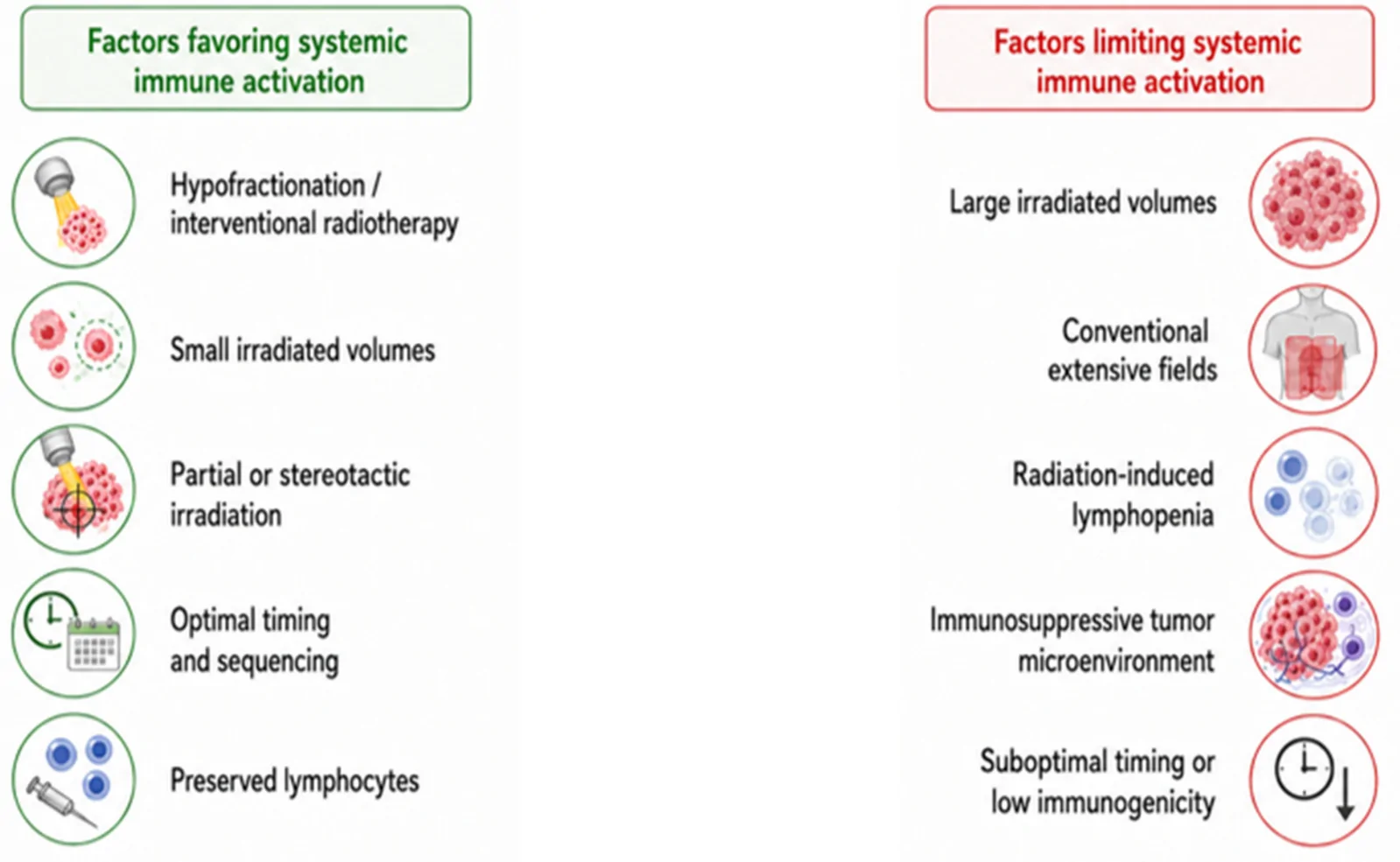
RT-related determinants of abscopal response in I-RT. See main text for details.

Emerging evidence also supports the potential role of IRT within I-RT strategies. The highly conformal nature of IRT allows delivery of ablative doses while minimizing irradiation of circulating immune cells and surrounding lymphoid structures [23,24]. This may theoretically enhance immune preservation and favor systemic responses. Figure 2 summarizes the principal RT-related factors that may promote or limit systemic immune activation.

Overall, the gap analysis revealed that RT, immunology, and clinical oncology remain weakly integrated domains. RT studies frequently neglect immune biomarkers, immunological investigations often lack clinically relevant dosimetric parameters, and clinical reports rarely include mechanistic interpretation or standardized translational endpoints. The main determinants of the abscopal effect, together with supporting evidence, translational gaps, and future research directions, are summarized in Table 1.

**Table 1:** Multidisciplinary determinants, representative evidence, translational gaps, and future directions in the abscopal effect.

Domain	Main Determinants	Representative Evidence	Translational Gap	Future Direction
RT	Dose, fractionation, target volume, sequencing, IRT	[16,17,23,24]	RT protocols are mainly optimized for local control rather than immune activation	Immune-adapted RT strategies integrating hypofractionation, smaller volumes, lymphocyte preservation, and optimal sequencing
Immunology	Immunogenic cell death, DAMPs, cGAS-STING, dendritic-cell activation, T-cell priming	[2,4,9,15]	Immune mechanisms are often studied without clinically relevant RT parameters	Longitudinal immune monitoring integrated with dosimetry and clinical outcomes
Clinical response	Tumor type, patient selection, timing of immunotherapy, toxicity, survival	[10,11,12,13,18,25]	Clinical evidence is heterogeneous and often lacks mechanistic interpretation	Biomarker-driven prospective trials with standardized translational endpoints
Tumor microenvironment	PD-L1, Tregs, MDSCs, immune-cell infiltration, lymphopenia	[7,9,17,22]	Limited ability to predict immune activation versus immune suppression after RT	Integration of TME profiling into immuno-radiotherapy planning
Extracellular vesicles	EV cargo, bystander signaling, immune modulation, liquid biopsy	[26,27,28,29]	Few studies link EVs with dose gradients, immune response, and abscopal-like effects	EV-based biomarkers integrated with RT dosimetry and immune monitoring
In vitro models	3D models, co-cultures, conditioned medium transfer, ROS, EV analysis	[30,31,32,33]	Current models are mostly 2D and poorly integrated with dosimetry and immune endpoints	Translational platforms combining 3D models, immune cells, EVs, and precise dose mapping
Advanced technologies	Imaging, radiomics, AI, FTIR spectroscopy, multimodal data integration	[26,27,34]	Lack of validated models combining imaging, biology, and dosimetry	AI-assisted frameworks for patient stratification and treatment personalization

Abbreviations: DAMPs, damage-associated molecular patterns; EVs, extracellular vesicles; FTIR, Fourier-transform infrared spectroscopy; IRT, interventional radiotherapy; MDSCs, myeloid-derived suppressor cells; PD-L1, programmed death-ligand 1; ROS, reactive oxygen species; RT, radiotherapy; TME, tumor microenvironment; Tregs, regulatory T cells.

## 4. Discussion

The discrepancy between strong preclinical evidence and inconsistent clinical outcomes likely derives from differences between experimental conditions and real-world clinical practice. Preclinical models often involve small irradiated volumes, controlled immune environments, and hypofractionated schedules specifically optimized to stimulate immune activation. In contrast, clinical treatments, particularly in locally advanced tumors such as head and neck cancer, frequently require extensive target volumes and conventional fractionation capable of inducing profound lymphopenia and systemic immune suppression. Importantly, the relationship between irradiated volume and immune response may also depend on radiation dose. While limiting irradiated volumes may be particularly relevant in the context of high-dose irradiation, emerging strategies are investigating whether low-dose irradiation of larger volumes could favorably modulate the tumor immune microenvironment. However, the clinical relevance of this approach remains to be established, and further preclinical and translational evidence is required to define the optimal interaction between dose and irradiated volume [35]. A related investigational approach combines hypofractionated high-dose irradiation of selected lesions with low-dose irradiation of other tumor sites. This strategy aims to exploit the potentially complementary immunological effects of different dose levels; however, its clinical benefit and the optimal dose and target selection remain to be established.

These observations suggest that RT should not merely be combined with immunotherapy but should instead be biologically tailored to immune-mediated mechanisms. The concept of “adapting RT to immunotherapy” has recently emerged as a central paradigm in modern I-RT [2]. Within the paradigm of immune-adapted RT, multiple treatment-related variables, including fractionation, irradiated volume, sequencing, tissue radiosensitivity, and target selection, may critically influence systemic immune activation. Among these variables, target selection remains one of the least investigated yet potentially most consequential determinants of abscopal responses. In metastatic disease, it remains unclear whether the primary tumor or a metastatic lesion constitutes the most effective immunological target. While the primary tumor may provide a broader antigen reservoir and harbor a more complex immune microenvironment, selected metastases may be more suitable for ablative irradiation and potentially exhibit lower levels of immune suppression. Furthermore, metastatic lesions are not biologically equivalent but represent distinct evolutionary states with different clonal and immune characteristics. Therefore, lesion selection in patients with multiple metastases should not be empirical, as local treatment may impose selective pressures that influence the subsequent evolution of the disease. Parameters such as lesion size, anatomical location, proximity to lymphoid structures, metastatic burden, vascularization, and local immune composition may all affect the likelihood of eliciting systemic anti-tumor responses. Identifying the optimal lesion for irradiation may therefore prove to be as important as optimizing dose, fractionation, and sequencing strategies, and should become a dedicated focus of future I-RT trials [8,36,37,38,39].

Beyond the selection of an individual target lesion, the number of metastatic sites irradiated may also influence systemic immune activation. Irradiation of multiple lesions may increase the overall release of tumor-associated antigens and expose a broader repertoire of tumor clones, potentially enhancing immune priming compared with single-lesion irradiation. However, this potential benefit should be balanced against the risk of increasing the irradiated volume and treatment-related lymphopenia. In this context, preservation of tumor-draining and regional lymph nodes may be particularly relevant, as these structures represent key sites for antigen presentation and T-cell priming. Unnecessary irradiation of uninvolved lymphatic regions could therefore impair the development of an effective systemic antitumor immune response. Future I-RT strategies should consider not only which lesion to irradiate, but also the number of lesions treated and the possibility of preserving immunologically relevant lymphatic structures whenever oncologically appropriate [40,41].

**Figure 3:**
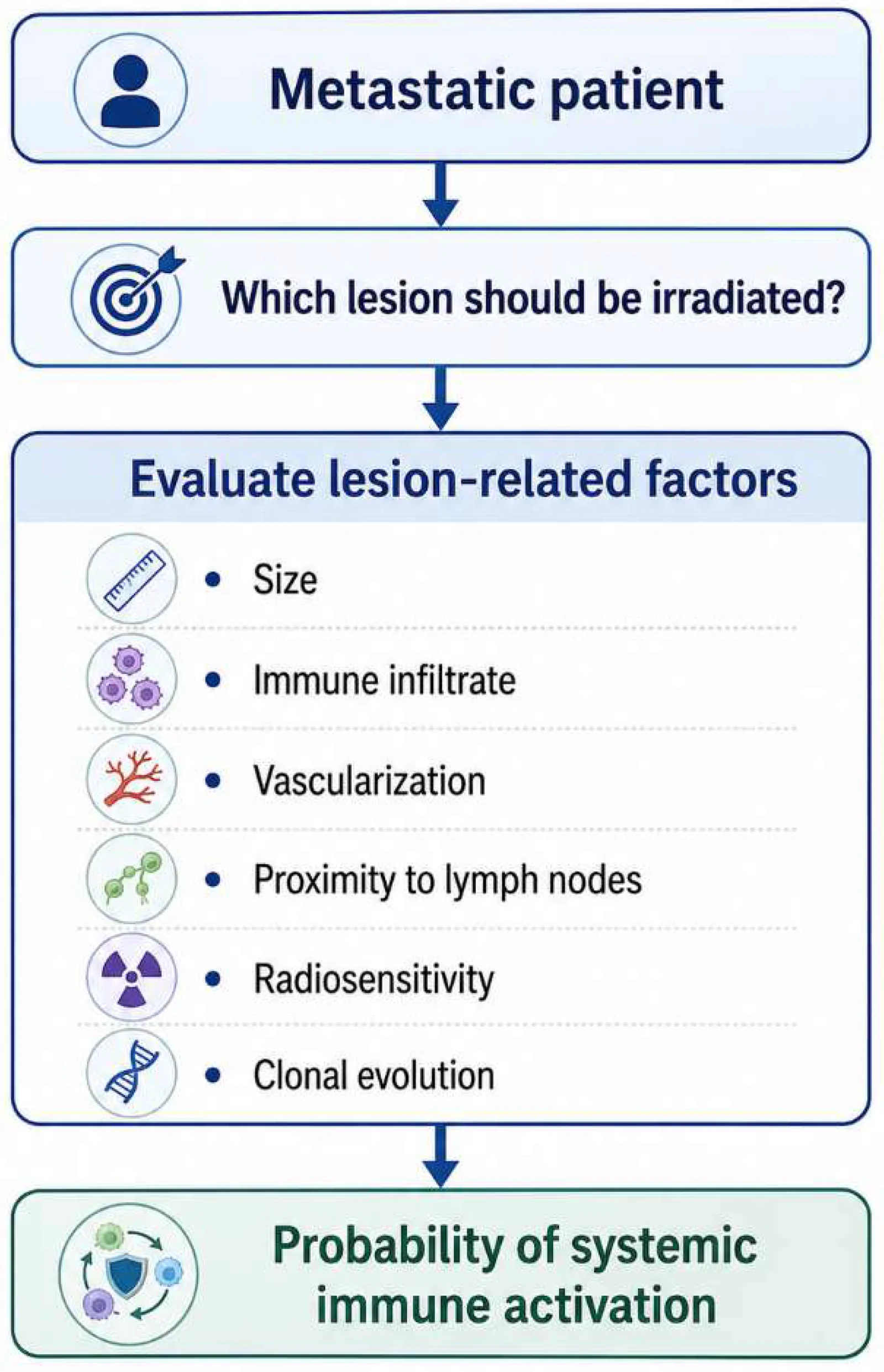
Conceptual framework for lesion selection in metastatic disease. See main text for details.

Because lesion selection may critically influence the likelihood of systemic immune activation, a schematic overview of the main variables involved is provided in Figure 3.

Hypofractionation appears particularly promising because it may enhance immunogenic cell death and preserve immune functionality more effectively than conventional fractionation [23]. However, the immunological relevance of radiation-induced cell death should not be interpreted only in terms of tumor antigen release. Although antigen availability is necessary, it is unlikely to be sufficient to generate effective systemic anti-tumor immunity in the absence of appropriate danger signals and antigen-presenting cell activation [3,15]. Radiation can promote the release or exposure of damage-associated molecular patterns, including calreticulin, HMGB1, ATP, cytosolic DNA, and type I interferon-related signals, which contribute to dendritic cell recruitment, maturation, antigen uptake, and cross-presentation. Therefore, the ability of RT to convert the irradiated lesion into an in situ vaccine may depend less on antigen release alone and more on the coordinated induction of immunogenic stress signals capable of activating APCs and initiating productive T-cell priming [6,9,15,42]. Similarly, partial tumor irradiation has emerged as a potential strategy to induce favorable immunological responses while limiting the overall irradiated volume, particularly in large or bulky lesions. Irradiation of selected tumor subvolumes may promote immunogenic signaling and tumor antigen release while reducing lymphocyte exposure; however, the optimal dose and target subvolume remain to be established [43].

In addition to fractionation, radiation dose represents a critical determinant of systemic immune activation. However, the optimal dose for eliciting the abscopal effect has not yet been established. Experimental studies suggest that intermediate ablative doses may maximize immunogenic cell death and activation of the cGAS–STING pathway while avoiding excessive induction of DNA exonucleases, such as TREX1, which may attenuate immune signaling after very high single doses. These observations indicate that the immunological effects of RT are not simply dose-dependent but result from the complex interaction among dose, fractionation, irradiated volume, and the tumor immune microenvironment. Consequently, identifying the optimal biological dose for promoting systemic antitumor immunity remains an important objective for future translational and clinical studies [42]. Emerging radiation-delivery approaches, including FLASH radiotherapy and spatially fractionated radiotherapy (SFRT), are also being investigated for their potential to modify the balance between tumor control, normal-tissue sparing, and immune modulation [44,45]. Early preclinical and clinical observations are encouraging; however, their specific contribution to systemic immune activation and abscopal responses remains to be established. Particle radiotherapy, particularly proton and carbon-ion therapy, represents another emerging area of interest in I-RT. Their distinct physical and radiobiological properties may offer advantages in limiting normal-tissue irradiation while potentially influencing tumor immunogenicity and immune modulation. However, evidence regarding their ability to enhance systemic antitumor immunity or abscopal responses remains limited, and further translational and clinical investigation is required [46]. Systemic targeted radioligand therapy (TRT) represents another emerging strategy with potential for combination with immunotherapy. By selectively delivering radiation to tumor-associated molecular targets, TRT may induce immunogenic effects across multiple disease sites and provide a rationale for combination with immune checkpoint inhibitors. Early clinical experience is encouraging; however, the optimal radiopharmaceutical, dose, sequencing, and patient selection remain to be established [47].

Another major determinant of radiation-induced systemic immunity is the tumor microenvironment. Although RT can promote immune activation through increased antigen presentation, T-cell infiltration, and type I interferon signaling, it may simultaneously induce compensatory immunosuppressive mechanisms. Radiation-induced upregulation of PD-L1, recruitment of regulatory T cells (Tregs), accumulation of myeloid-derived suppressor cells (MDSCs), and macrophage polarization may all counteract effective anti-tumor immunity. Consequently, the balance between immune stimulation and immune suppression within the irradiated lesion may critically determine whether local treatment generates productive systemic responses or reinforces immune escape. This dual nature of RT suggests that the biological context of the tumor microenvironment, including pre-existing immune infiltration and the distinction between immune-hot and immune-cold tumors, may represent an additional layer of complexity influencing the probability of observing abscopal effects. Integrating tumor microenvironment profiling into I-RT strategies may therefore contribute to a more personalized and biologically informed approach. Two non-mutually exclusive mechanisms may therefore limit the development of abscopal responses. First, local RT may simultaneously induce immunostimulatory and immunosuppressive signals, thereby attenuating effective immune priming. Second, even when tumor-specific adaptive immunity is successfully generated, its activity may be restrained within distant non-irradiated lesions by pre-existing immunosuppressive microenvironments. These scenarios have different therapeutic implications, supporting either optimization of RT parameters to favor immune activation or combination strategies aimed at overcoming immune resistance at distant metastatic sites [7,9,48]. Tumor histology may further influence the efficacy of I-RT. Tumors such as melanoma and selected lung cancers frequently exhibit higher tumor mutational burden, greater neoantigen availability, and a more pre-existing immune-infiltrated microenvironment, potentially facilitating radiation-induced immune priming and responsiveness to immune checkpoint inhibition. Conversely, immune-cold tumors or tumors characterized by strong stromal and immunosuppressive barriers may be less likely to develop systemic immune responses following local RT. These differences suggest that I-RT should not necessarily be restricted to specific tumor types, but rather guided by biological and immunological features capable of identifying patients most likely to benefit.

Immune monitoring represents another important but still insufficiently standardized component of I-RT studies. Analysis of the irradiated lesion, distant non-irradiated lesions, and peripheral blood may provide complementary information on local and systemic immune responses. In particular, circulating lymphocyte subsets have shown potential associations with treatment response; however, their predictive value in I-RT remains insufficiently validated [49]. Further preclinical and in vitro studies integrating tissue and peripheral immune readouts with clinically relevant radiation parameters are therefore needed before specific monitoring strategies can be established.

The role of IRT deserves particular attention. IRT enables highly localized dose delivery with steep dose gradients and reduced irradiation of surrounding normal tissues and lymphatic structures. This characteristic may theoretically preserve circulating lymphocytes and facilitate local immune activation while minimizing systemic immune suppression [50]. Furthermore, high-LET radiation approaches such as alpha-emitter therapies may promote a stronger induction of antigen release and immunogenic tumor cell death.

Although radiotherapy remains the most extensively investigated modality in the context of the abscopal effect, increasing evidence suggests that other ablative techniques may also elicit systemic immune responses. Non-thermal approaches, such as irreversible electroporation (IRE), induce tumor cell death while preserving the extracellular matrix and vascular structures, potentially facilitating immune cell infiltration. Histotripsy mechanically disrupts tumor tissue using focused ultrasound without thermal injury and has recently demonstrated promising immunostimulatory properties in preclinical models. Thermal ablative techniques, including microwave ablation (MWA) and radiofrequency ablation (RFA), induce coagulative tumor necrosis accompanied by the release of tumor antigens, DAMPs, and pro-inflammatory mediators that may promote systemic immune activation. Among radiation-based ablative strategies, stereotactic body radiotherapy (SBRT) delivers highly conformal, high-dose radiation in a limited number of fractions, achieving local tumor ablation while minimizing irradiation of surrounding healthy tissues. Owing to its ability to induce immunogenic cell death while limiting irradiation of normal tissues and circulating immune cells, SBRT has emerged as one of the most promising radiotherapy approaches for combination with I-RT and for enhancing the likelihood of abscopal responses. Although clinical evidence supporting true abscopal responses remains limited across these modalities, their integration with immunotherapy represents an active area of investigation and may further expand the therapeutic potential of local ablative treatments. The main characteristics of these ablative techniques, together with their proposed immunological mechanisms and the current evidence supporting abscopal-like responses, are summarized in Table 2.

Recent reports have also described the so-called badscopal effect, referring to the unexpected progression of non-irradiated metastatic lesions following local radiotherapy. Current evidence remains limited, and the underlying mechanisms have not yet been fully elucidated. Proposed explanations include unfavorable immune modulation, treatment-related lymphopenia, and transient systemic immunosuppression. Rather than contradicting the biological plausibility of the abscopal effect, these observations highlight the complexity of radiation-induced systemic immune responses and further emphasize the importance of optimizing radiation dose, target volume, fractionation, and treatment sequencing to maximize beneficial immune-mediated effects while minimizing potentially adverse systemic consequences [51].

Treatment sequencing represents another critical determinant. Emerging evidence suggests that neoadjuvant integration of immunotherapy may produce stronger immune priming than adjuvant approaches [21]. However, the optimal temporal relationship between RT and immune checkpoint inhibition remains incompletely defined.

Artificial intelligence, radiomics, and advanced imaging may help overcome several of the current limitations. Imaging biomarkers capable of capturing spatial and temporal patterns of immune activation could facilitate patient selection and response prediction. Computational approaches integrating dosimetric, biological, and clinical data may additionally help identify personalized treatment strategies. AI-driven integration of imaging, dosimetry, immune profiling, and circulating biomarkers may facilitate the identification of patient-specific signatures associated with immune activation and treatment response [52].

A further translational opportunity is represented by the development of in vitro platforms specifically designed to investigate the abscopal effect under controlled radiobiological conditions. Although clinical abscopal responses are difficult to isolate from the effects of systemic therapies and tumor heterogeneity, in vitro models can help dissect the contribution of radiation-induced bystander signaling, oxidative stress, immune activation, and soluble mediators released from irradiated cells. The integration of 3D-printing technologies may further enhance treatment personalization by enabling the development of patient-specific applicators, templates, and dosimetric phantoms tailored to individual anatomy and treatment geometry. In parallel, the use of biocompatible biomaterials for 3D-printed platforms may offer additional opportunities for developing customized experimental systems with favorable biological and immunological compatibility [53,54].

In this context, future models should not focus exclusively on tumor antigen release, but should also evaluate the generation of danger signals and the downstream activation of antigen-presenting cells. Endpoints such as calreticulin exposure, HMGB1 and ATP release, cGAS-STING activation, type I interferon signaling, dendritic cell maturation markers, cytokine secretion, antigen uptake, and cross-presentation capacity may provide a more complete assessment of the immunogenic potential of irradiated tumor cells [2,3,6]. This is particularly relevant for IRT, where steep dose gradients may generate spatially heterogeneous biological stress within the same experimental system. Current HDR IRT in vitro research remains limited by the predominance of simplified 2D cultures, scarce use of spheroids or organoids, limited immune-related endpoints, and insufficient integration between biological readouts and high-precision dosimetry. Therefore, future experimental models aimed at studying the abscopal effect should incorporate multicellular co-cultures, immune-cell components, conditioned-medium transfer experiments, extracellular vesicle analysis, ROS quantification, danger signal profiling, APC activation assays, dendritic cell maturation and cross-presentation readouts, and 3D dose mapping, allowing a more realistic evaluation of how localized irradiation may trigger distant immune-mediated responses. These platforms may also provide a controlled environment to investigate dose-dependent immune activation and dissect the relative contribution of antigen release, danger signaling, extracellular vesicles, and bystander effects to systemic responses [30].

**Figure 4:**
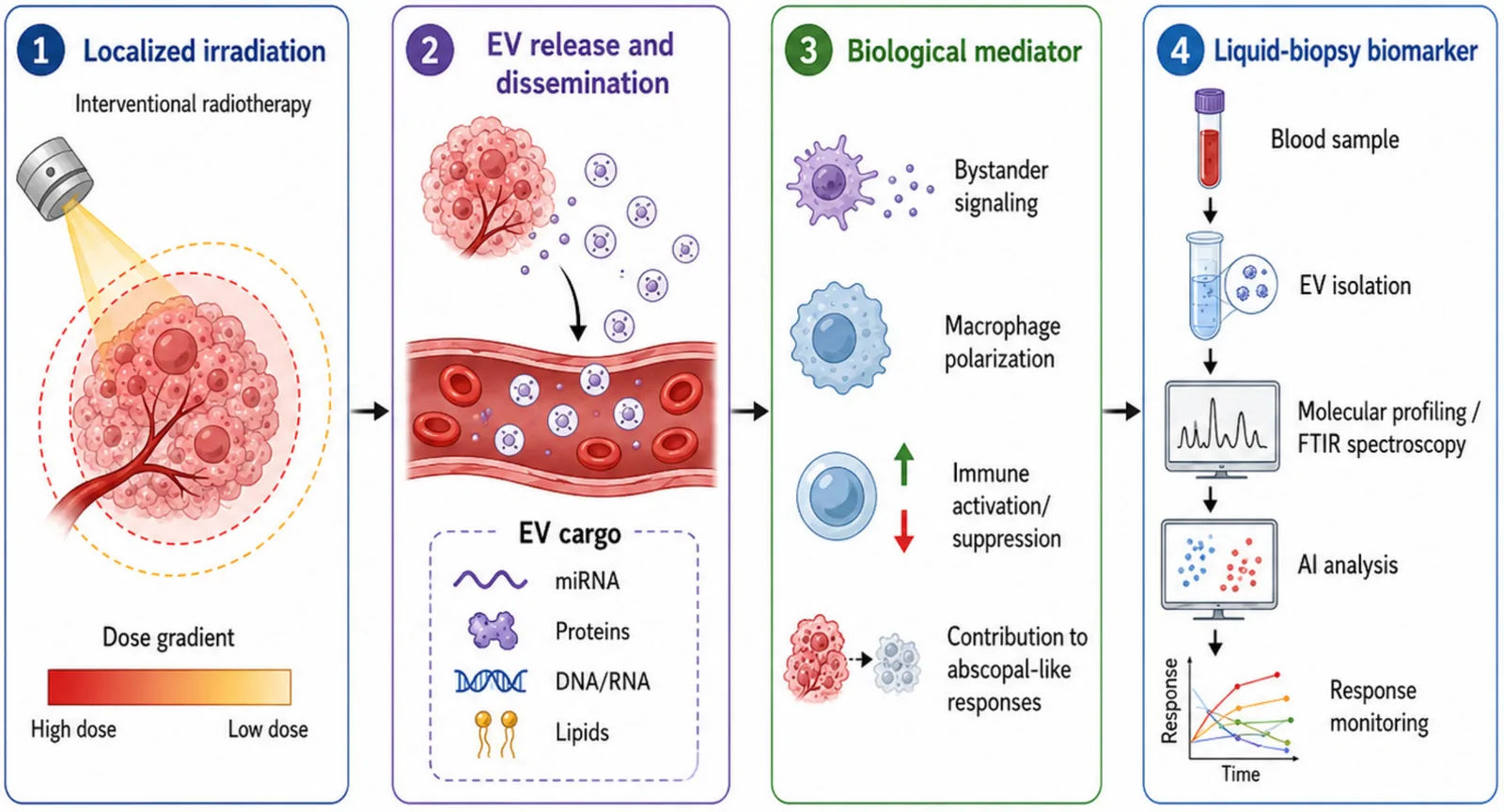
Extracellular vesicles as mediators and liquid-biopsy biomarkers. See main text for details.

Taken together, these observations suggest that the probability of generating abscopal responses does not depend on a single variable, but rather on the dynamic interaction among radiation parameters, tumor microenvironment, immune activation, and biological mediators acting across local and systemic compartments. Extracellular vesicles represent one of the most promising emerging biomarkers and mediators of radiation-induced immune response. EVs carry proteins, lipids, nucleic acids, and metabolites that reflect the biological state of their cells of origin, and their cargo can change after irradiation. In the context of radiobiology, EVs may represent a measurable interface between local radiation-induced damage and systemic biological communication, including bystander effects, immune activation or suppression, macrophage polarization, cytokine-related signaling, and treatment resistance. Their accessibility in biological fluids makes them particularly attractive as liquid biopsy biomarkers for monitoring IRT responses. Recent work using Fourier-transform infrared (FTIR) spectroscopy, a vibrational spectroscopic technique that characterizes the biochemical composition of biological samples, and autoencoder-based analysis demonstrated that EV biochemical fingerprints can be transformed into biologically meaningful latent features with diagnostic potential, supporting the possibility that EV-based profiles may capture complex treatment-related or disease-related biological states beyond single-marker assays [26,55]. This concept may be particularly relevant for IRT, whose steep spatial dose gradients may induce heterogeneous EV release from highly irradiated, sublethally irradiated, and non-irradiated neighboring compartments. In such a model, EVs could act both as reporters of local dose-dependent stress and as mediators of paracrine or systemic signaling. Although preliminary evidence suggests that IRT can modulate EV populations and cargo, the available studies remain few, heterogeneous, and rarely designed to link EV signatures with spatial dose–response relationships or immune endpoints. Integrating EV characterization with IRT-specific dosimetry could therefore provide a translational framework to connect physical dose gradients, radiobiological response, and systemic immune effects, potentially improving the interpretation of abscopal-like mechanisms [27,56,57,58]. This proposed framework is summarized in Figure 4.

Importantly, the rarity of clinically documented abscopal responses should not necessarily be interpreted as evidence of biological insignificance. Rather, it likely reflects the complexity of systemic anti-tumor immunity and the absence of standardized multidisciplinary frameworks capable of integrating radiobiological, immunological, and clinical information. This challenge is further compounded by the lack of evidence-based criteria for selecting the optimal irradiation target in metastatic disease, a variable that may critically influence the likelihood of eliciting systemic immune responses and abscopal effects.

## 5. Conclusions

The abscopal effect remains an important translational topic in contemporary oncology. Although localized RT can contribute to systemic immune-mediated responses, its clinical reproducibility is still limited. This review suggests that a key barrier is the fragmentation of evidence across RT, immunology, and clinical oncology. Better integration of dosimetric parameters, immune mechanisms, biomarkers, and clinical outcomes may help clarify the conditions under which abscopal responses are more likely to occur. Future studies should focus on translational frameworks linking radiation parameters, immune monitoring, extracellular vesicle-based biomarkers, and clinical response. This integrated approach may support a more consistent interpretation of the abscopal effect within I-RT.

## Figures and Tables

**Table 2:** Comparison of local ablative techniques and their potential relationship with the abscopal effect.

Technique	Mechanism of Action	Immunological Effects	Evidence for Abscopal Effect
IRT	High-dose localized radiation delivered with steep dose gradients	Immunogenic cell death, antigen release, DAMP release, preservation of surrounding tissues	Emerging preclinical and limited clinical evidence
SBRT	High-dose external beam radiation in a few fractions	Immunogenic cell death, cGAS–STING activation, T-cell priming	Most extensive clinical evidence among radiotherapy-based ablative approaches, particularly in combination with I-RT.
Irreversible electroporation (IRE)	Non-thermal membrane permeabilization	Preserves extracellular matrix, facilitates immune infiltration	Preclinical evidence and early clinical reports
Histotripsy	Mechanical tissue disruption using focused ultrasound	Release of tumor antigens with minimal thermal damage	Promising preclinical evidence; clinical evidence emerging
Microwave ablation (MWA)	Thermal coagulative necrosis	Antigen release and pro-inflammatory cytokine production	Limited clinical reports; mainly combined with immunotherapy
Radiofrequency ablation (RFA)	Thermal coagulative necrosis	Antigen release, dendritic-cell activation, inflammatory signaling	Case reports and small clinical series suggest occasional abscopal responses

Abbreviations: cGAS–STING, cyclic GMP–AMP synthase–stimulator of interferon genes; I-RT, immune-radiotherapy.

## Data Availability

No new data were created or analyzed in this study. Data sharing is not applicable to this article.
